# The dose makes the poison: Mechanisms of cytotoxicity of high-dose AAV vectors

**DOI:** 10.1016/j.omta.2026.201746

**Published:** 2026-05-09

**Authors:** Terence R. Flotte

**Affiliations:** 1 Department of Genetic and Cellular Medicine and Pediatrics, UMass Chan Medical School, Worcester, MA 01520, USA

## Main text

In the year 1538, the Swiss physician Paracelsus wrote, “All things are poison, and nothing is without poison; the dosage alone makes it so a thing is not a poison.”^1^ Recombinant adeno-associated virus (rAAV) vectors have long been known for their favorable safety profile. However, in recent years, as manufacturing technology improved, the maximum feasible doses of rAAV increased and a small percentage of patients have experienced severe and even fatal toxicities from rAAV gene therapy. Several different toxicity syndromes have emerged, including hepatotoxicity, thrombotic microangiopathy (TMA), thrombocytopenia, myocarditis, acute respiratory distress syndrome, and hemophagocytic lymphohistiocytosis (HLH).^2^^,^^3^ None of these empirically observed syndromes were predicted prior to being observed in humans, and the cellular and molecular basis for these syndromes is not well understood.

In this issue of *Molecular Therapy Advances*, Moeini et al. examine the roles of unfolded protein responses (UPRs), DNA damage/p53 responses, innate immune and liver metabolic pathways in hepatotoxicity following high-dose systemic rAAV9-SMN1 administration in two different species, non-human primates (NHPs, cynomolgus macaques) and rats.^4^ Expression profiling by RNA sequencing was used to determine the relative mRNA levels at various doses across the two species, showing gene expression profiles characterizing activation or repression of specific genes or pathways. Differentially expressed genes were identified and categorized in each of two standard systems, the Molecular Signature Database and the Gene Ontology Database.

The responses to increasing vector dose were somewhat distinct in the two species. Rats did not express the transgene as robustly and lacked induction of the UPR. This strongly suggests that UPR-related changes were specifically related to the synthesis of transgene product. Across all the datasets, the magnitude of UPR activation, particularly the PERK-induced (endoplasmic reticulum [ER] stress) response, was strongly associated with the level of transgene expression. Genes involved in triggering mitochondrial apoptosis were also induced by this pathway. This same set of conditions repressed the expression of XBP1s or processed ATF6 proteins, which could have a pro-survival function. The activation of immune responsive pathways and the decrease in expression of liver metabolic genes, which were taken as “liver identity” genes for this purpose, were seen across both species and both sexes of animals.

**Figure 1:**
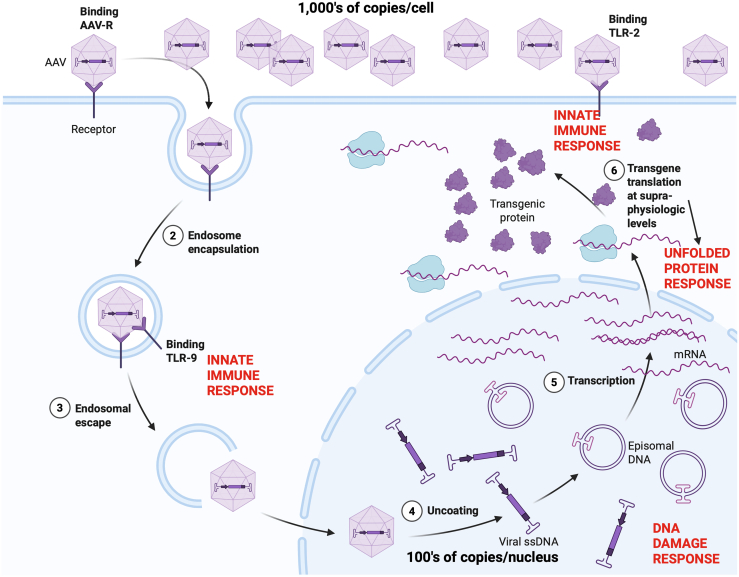
Cellular events triggered by exposure to high systemic doses of rAAV vectors Created with BioRender, license obtained.

These findings fit into a broader picture that has been evolving over the past three decades about how rAAV vector DNA reacts to host cell factors at the molecular level. It is important to keep in mind what rAAV vectors consist of: a single-stranded DNA of 4.7kb in length, presented to cells at copy numbers reaching well into the thousands (Figure 1). Once AAV vector DNA enters the nucleus, it is converted to a double-stranded form and thereby presents the nuclear environment with hundreds of copies of what appear to be double-stranded DNA breaks. Activation of double-strand break repair has been identified in cell culture and *in vivo*.^5^^,^^6^^,^^7^ The interactions with other host pathways are complex and may lead to various results, including apoptosis in cells that lack p53 expression.^7^

While not all rAAV vector copies enter the nucleus, those within endosomes and lysosomes and at the cell membrane can also have important effects on host pathways including through activation of Toll-like receptor (TLR) 2 and TLR9.^8^ The activation of toll receptors is an initial step in activation of various innate immune response genes, including cytokine encoding genes.

Finally, once the vector DNA becomes transcriptionally active, it often mediates massive overexpression of the transgene product, as strong constitutively active promoters continue to be frequently used. These levels of overexpression can lead to UPR activation, as the authors of this paper demonstrate. This form of dose-related toxicity has been observed in the dorsal root ganglia and is less significant when the transcriptional activity is lower.^9^^,^^10^

It is important to note that these multiplicities of infection would never be encountered in nature, so the lessons to be drawn from natural AAV infection are quite limited. Thus, exposing humans to very high doses of rAAV vectors may, in fact, turn a benign non-pathogenic virus into a toxic substance, as Paracelsus might have predicted.

The only way to improve the safety of these vectors in humans is to conduct translational research both forward and backward, attempting to reproduce aspects of AAV vector toxicity in animal models where the molecular mechanisms that underlie them can be more carefully studied. This paper by Moeini is an important example of the “backward translation” process. Future research of this type, while less exciting than bringing forward yet another potential cure, may in the long run benefit more patients than would any one new vector brought to the clinic. Only through such studies can the full potential of AAV vector technology be unlocked to maximize its impact on human diseases.

## Declaration of interests

The authors declare no competing interests.

## References

[1] Paracelsus (1538).

[2] Duan D. (2023). Lethal immunotoxicity in high-dose systemic AAV therapy. Mol. Ther..

[3] Byrne B.J., Flanigan K.M., Matesanz S.E., Finkel R.S., Waldrop M.A., D'Ambrosio E.S., Johnson N.E., Smith B.K., Bönnemann C., Carrig S. (2025). Current clinical applications of AAV-mediated gene therapy. Mol. Ther..

[4] Moeini P., Bilbao-Arribas M., Guruceaga E., Torrens-Baile J., Lanz T.A., Whiteley L.O., Aragón T., Unzu C., González-Aseguinolaza G. (2026). DNA damage/p53, innate immune, and unfolded protein responses are activated in primate liver after toxic, high-dose AAV-SMN1 delivery. Molecular Therapy Advances.

[5] Song S., Laipis P.J., Berns K.I., Flotte T.R. (2001). Effect of DNA-dependent protein kinase on the molecular fate of the rAAV2 genome in skeletal muscle. Proc. Natl. Acad. Sci. USA.

[6] Schwartz R.A., Carson C.T., Schuberth C., Weitzman M.D. (2009). Adeno-associated virus replication induces a DNA damage response coordinated by DNA-dependent protein kinase. J. Virol..

[7] Raj K., Ogston P., Beard P. (2001). Virus-mediated killing of cells that lack p53 activity. Nature.

[8] Cao D., Byrne B.J., de Jong Y.P., Terhorst C., Duan D., Herzog R.W., Kumar S.R.P. (2024). Innate Immune Sensing of Adeno-Associated Virus Vectors. Hum. Gene Ther..

[9] Hordeaux J., Hinderer C., Goode T., Buza E.L., Bell P., Calcedo R., Richman L.K., Wilson J.M. (2018). Toxicology Study of Intra-Cisterna Magna Adeno-Associated Virus 9 Expressing Iduronate-2-Sulfatase in Rhesus Macaques. Mol. Ther. Methods Clin. Dev..

[10] Hordeaux J., Buza E.L., Dyer C., Goode T., Mitchell T.W., Richman L., Denton N., Hinderer C., Katz N., Schmid R. (2020). Adeno-Associated Virus-Induced Dorsal Root Ganglion Pathology. Hum. Gene Ther..

